# A multicenter multifunctional assessment of large language models in pure-tone audiogram interpretation for patients

**DOI:** 10.1038/s41746-026-02537-1

**Published:** 2026-03-15

**Authors:** Jun Liang, Mengyao Xing, Peng Xiang, Guixuan Wang, Ming Chen, Qichuan Fang, Tingting Zhou, Zhengyang Lu, XueMing Leng, Jiuke Huang, Xiaoyi Jiao, Chenghua Tian, Jianbo Lei

**Affiliations:** 1 Department of AI and IT, Second Affiliated Hospital, School of Medicine, Zhejiang University, Hangzhou, China; 2 School of Medical Technology and Information Engineering, Zhejiang Chinese Medical University, Hangzhou, China; 3 School of Public Health, Zhejiang University, Hangzhou, China; 4 National Key Laboratory of Transvascular Implantable Devices, Second Affiliated Hospital, School of Medicine, Zhejiang University, Hangzhou, China; 5 Intelligent Medical Research Center, Zhejiang University Institute of Computer Innovation Technology, Hangzhou, China; 6 Department of Otolaryngology, Second Affiliated Hospital, School of Medicine, Zhejiang University, Hangzhou, China; 7 School of Nursing, Zhejiang Chinese Medical University, Hangzhou, China; 8 School of Electronic, Electrical and Communication Engineering, University of Chinese Academy of Sciences, Beijing, China; 9 Department of Computer Science, Columbia University in the City of New York, New York, NY USA; 10 Clinical Research Center, Affiliated Hospital of Southwest Medical University, Luzhou, China; 11 School of Medical Information and Engineering, Southwest Medical University, Luzhou, China; 12 Center for Medical Informatics, Institute of Advanced Clinical Medicine, Peking University, Beijing, China

**Keywords:** Health care, Medical research, Signs and symptoms

## Abstract

No LLMs (Large Language Models) have yet been evaluated for understanding picture reports. Pure-tone audiograms, the gold standard for hearing loss assessment, are technical and often incomprehensible to patients without specialist interpretation. We conducted a blinded, multicenter evaluation of eight LLMs across diagnostic, interpretive, and recommendation tasks using 140 audiogram reports, assessed by clinicians and lay reviewers. The study revealed that DeepSeek-V3 achieved the highest diagnostic accuracy (severity: 67.00% ; type: 54.00%), R1 proved most suitable for general readership (FKGL: 6.41). The general public perceived significant benefits from all models in comprehension and emotional support, with Gemini 2.0 Flash/Thinking scoring higher. Challenges remain in understanding pathological mechanisms and controlling hallucinations. While current general-purpose LLMs cannot replace the diagnostic capabilities of physicians, they may serve as effective auxiliary tools for translating specialized audiogram data into structured, patient-accessible interpretations, with particular relevance for populations facing limited access to hearing-care services.

## Introduction

As a globally prevalent health issue, hearing loss has been clearly defined as a significant public health concern^1–3^. The World Health Organization (WHO) estimates in its latest World Report on Hearing that nearly 2.5 billion people worldwide will experience some degree of hearing loss by 2050^4^. Hearing loss is now recognized as the most prevalent sensory organ disability worldwide^5^, ranking third in the Global Burden of Disease (GBD) Years Lived with Disability (YLD) list, surpassed only by low back pain and migraine, and first among sensory impairments. Pure Tone Audiometry (PTA) is the gold standard for assessing hearing loss and functional testing^6^. Pure tone audiogram reports, generated from PTA results, are hearing health care graphical reports documenting a subject’s auditory responses to pure tone signals of varying frequencies and intensities. They serve as critical evidence for assessing auditory function, determining the degree, type, and location of hearing loss, and are widely used in otologic disease diagnosis, occupational health monitoring, and other fields^7,8^.

Currently, hearing health services face a dual challenge: on one hand, specialized hearing resources remain relatively scarce globally; on the other hand, traditional pure-tone audiogram reports, with their highly technical charts and concise diagnostic conclusions, often lack specific guidance and explanations tailored to individual patients. This leaves most individuals struggling to accurately understand their own hearing status and unable to receive clear recommendations on improving communication abilities^9^, such as through hearing aid use. The emergence of LLMs presents a potential solution. LLMs possess the capability to process and understand human language, as well as generate coherent question-answering responses^10^, and have played a positive role in enhancing patient-centered healthcare services. LLMs play an increasingly critical role in enhancing patient care quality (e.g., clinical decision support^11^, patient communication, and health education^12^) and optimizing healthcare processes (e.g., intelligent triage and guidance^13,14^, medical record, and coding automation^15,16^, appointment scheduling and resource allocation^17^). Their effective evaluation is therefore paramount.

Existing studies have explored LLM applications in interpreting reports across multiple medical specialties, such as radiology^18^, ophthalmology^19,20^, laboratory medicine^21^, and pathology^22^. However, according to benchmarks and tasks compiled by Li et al.^23^, prior research has predominantly focused on text summarization (TS) and dialog generation (DG) tasks, with minimal attention paid to evaluating general-purpose large models on medical image captioning (IC) tasks. This study will conduct the first investigation of general LLMs in otolaryngology-head and neck surgery (OHS) for IC tasks, focusing on whether LLMs can comprehend pure-tone audiogram reports and generate accurate, logical, and linguistically sound hearing interpretations and personalized recommendations that provide practical assistance to patients. This will systematically evaluate their potential value in aiding patients’ understanding of their hearing status and supporting hearing health decisions.

## Results

### Accuracy of LLMs in hearing diagnosis and hallucinations

**Fig. 1:**
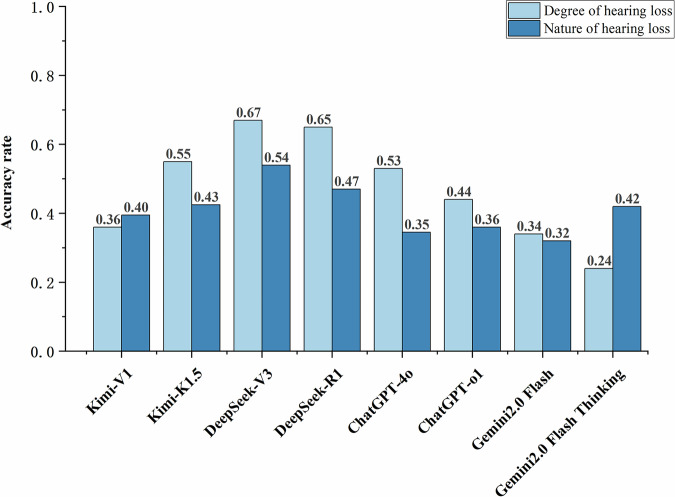
Diagnostic accuracy of LLMs in hearing test reports. This bar chart presents the accuracy rates of various models in classifying two aspects of hearing loss: degree of hearing loss (light blue) and nature of hearing loss (dark blue). Each line or point style corresponds to a distinct model, including open-source LLMs Kimi-V1, Kimi-K1.5, Deepseek-V3, Deepseek-R1, and closed-source LLMs ChatGPT-4o, ChatGPT-o3, Gemini 2.0 Flash, and Gemini 2.0 Flash Thinking.

The accuracy rates of the 8 LLMs models varied in hearing loss diagnosis. When diagnosing the degree of hearing loss based on provided audiograms (see Fig. 1, Supplementary Table 1), DeepSeek-V3 achieved the highest accuracy at 67.00%, followed by DeepSeek-R1 at 64.50%, while the remaining four models fell below 50%. Regarding the diagnosis of the type of hearing loss (e.g., conductive, sensorineural, or mixed), only DeepSeek-V3 achieved an accuracy rate above 50% (54.00%), while Gemini 2.0 Flash recorded the lowest rate (32.00%). Given the suboptimal diagnostic accuracy in the main experiments, this study conducted two supplementary experiments (see Supplementary Table 1). Results showed that diagnostic accuracy significantly improved (*p* < 0.01) for all six models except the two DeepSeek variants. Furthermore, the improvement in accuracy for diagnosing hearing severity was generally superior to that for diagnosing hearing loss type (*p* < 0.01).

Additionally, we quantified hallucinations in each LLMs’ output. Among 100 responses, both Kimi-V1 and ChatGPT-4o generated the highest number of hallucinatory reports (*n* = 24, 24%), while Gemini 2.0 Flash Thinking produced the fewest (*n* = 4, 4%). Detailed results are presented in Supplementary Fig. 1.

### Readability of LLM-generated content

Supplementary Table 2 presents the results of readability analysis for eight LLMs. From the FRE perspective, content generated by Kimi-V1 scored highest (66.05, 95% CI 63.08–68.90), while text produced by Kimi-K1.5 had the lowest FRE score (44.71, 95% CI 38.90–50.50). Regarding FKGL, DeepSeek generated the lowest average FKGL scores (V3: 6.83, 95% CI 6.40–7.30, R1: 6.41, 95% CI 5.93–6.98), while Kimi-K1.5 produced the highest FKGL scores (10.17, 95% CI 8.90–11.40). Regarding response length, Gemini 2.0 Flash produced the longest texts (21,885.54 words, 95% CI 20,864.00–22,899.25) among the eight LLMs, while DeepSeek-V3 generated the shortest texts (8,131.60 words, 95% CI 7675.50–8618.00) (Supplementary Fig. 2), with statistically significant differences (*p* < 0.01).

### Experts’ comprehensive evaluation of LLMs

**Fig. 2:**
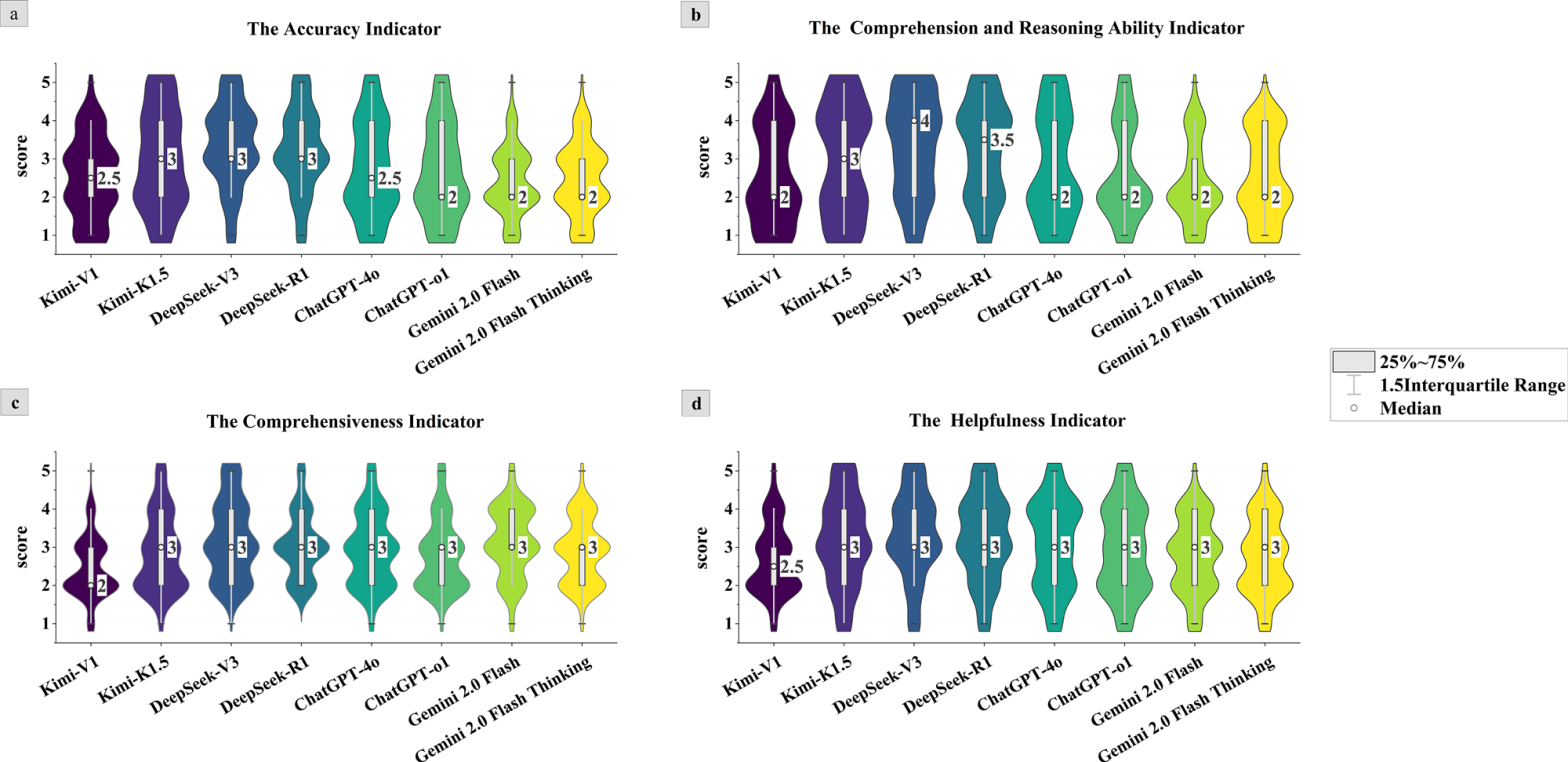
Results of expert evaluations of LLMs output text. The four panels (**a**–**d**) display violin plots comparing the following indicators across models: **a** The Accuracy Indicator, **b** The Comprehension and Reasoning Ability Indicator, **c** The Comprehensiveness Indicator, **d** The Helpfulness Indicator. Each plot shows the distribution of scores (with the median and interquartile range) for models including Kimi-V1, Kimi-K1.5, DeepSeek-V3, DeepSeek-R1, ChatGPT-4o, ChatGPT-01, Gemini2.0 Flash, and Gemini2.0 Flash Thinking. This violin plot displays the distribution of expert evaluation scores across different large language models. Each violin shape reflects the density distribution of the data.

Using human expert consensus assessment as the gold standard, we evaluated the performance of these eight models in diagnostic processes, interpretation, and recommendations. Additionally, we performed a Cohen’s kappa analysis on the consistency of expert assessments. The specific Kappa values are presented in Supplementary Table 3. Based on the evaluation thresholds by JR Landis and GG Koch^24^, the Cohen’s kappa for expert assessments falls within the range of strong agreement, indicating minimal variation in expert consensus and consistent reliability of the expert assessment metrics. Figures 2 and 4, along with Supplementary Table 4, illustrate the response characteristics of the eight models as assessed by experts. In overall accuracy evaluation, DeepSeek-V3 and R1 achieved higher average scores of 3.35 (95% CI 3.14–3.56, *p* < 0.01) and 3.22 (95% CI 3.00–3.43, *p* < 0.01), while Gemini2.0 Flash and 2.0Flash Thinking scored relatively lower at 2.38 (95% CI 2.21–2.55, *p* < 0.01) and 2.45 (95% CI 2.26–2.64, *p* < 0.01). Scores for the diagnostic reasoning section indicated that DeepSeek-V3 and R1 remained competitive, with average scores of 3.35 (95% CI 3.14–3.56, *p* < 0.01) and 3.24 (95% CI 3.00–3.43, *p* < 0.01), respectively, while Gemini 2.0 Flash scored the lowest (2.38, 95% CI 2.21–2.55, *p* < 0.01). Furthermore, the results of the comprehensive assessment of report interpretation indicated that DeepSeek-V3 and Gemini 2.0 Flash performed best, with average scores of 3.19 (95% CI 2.99–3.39, *p* < 0.01) and 3.14 (95% CI 2.96-3.32, *p* < 0.01), respectively. DeepSeek-R1 followed closely (3.08, 95% CI 2.91–3.25, *p* < 0.01), while Kimi-V1 received the lowest performance score (2.45, 95% CI 2.29–2.61, *p* < 0.01). Finally, the helpfulness assessment in the recommendation section showed DeepSeek-V3 scored highest (3.30, 95% CI 3.08–3.52, *p* < 0.01), while Kimi-V1 scored lowest (2.69, 95% CI 2.51–2.87, *p* < 0.01).

### Public evaluation

**Fig. 3:**
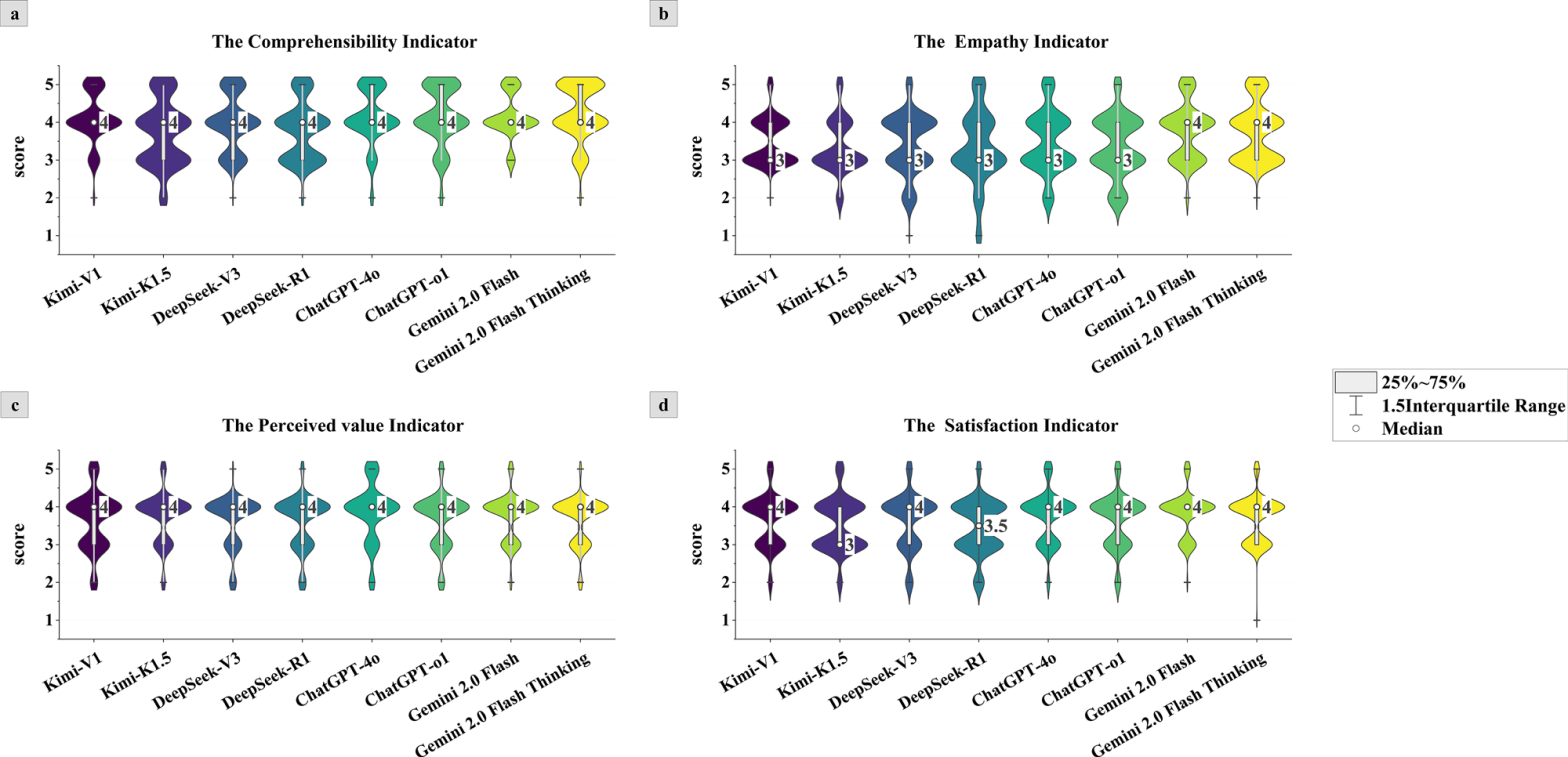
Public evaluation results of LLMs output text. The four panels (**a**-**d**) display violin plots comparing the following indicators across models: **a** The Comprehensibility Indicator, **b** The Empathy Indicator, **c** The Perceived value Indicator, **d** The Satisfaction Indicator. Each plot shows the distribution of scores (with the median and interquartile range) for models including Kimi-V1, Kimi-K1.5, DeepSeek-V3, DeepSeek-R1, ChatGPT-4o, ChatGPT-01, Gemini2.0 Flash, and Gemini2.0 Flash Thinking. This violin plot illustrates the distribution of public evaluation scores across various LLMs. Each violin shape reflects the density distribution of the data.

**Fig. 4:**
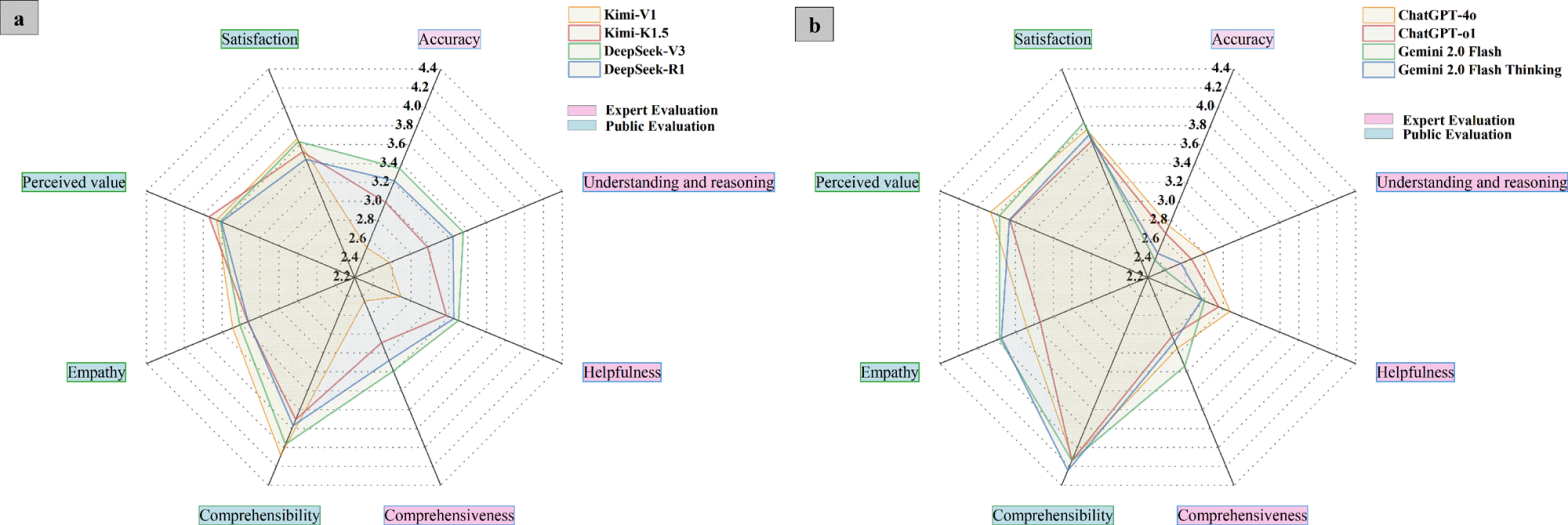
Radar chart of cross-model evaluation dimensions from expert and public perspectives. **a** the comprehensive performance evaluation results of China LLMs; **b** the comprehensive performance evaluation results of U.S. LLMs. The pink box indicates dimensions evaluated from the expert perspective, while the blue box represents dimensions evaluated from the public perspective. The plotted indicators include accuracy, understanding and reasoning, comprehensiveness, helpfulness, comprehensibility, empathy, perceived value, and satisfaction, with models such as Kimi-V1, Kimi-K1.5, DeepSeek-V3, DeepSeek-R1, ChatGPT-4o, ChatGPT-o1, Gemini2.0 Flash, and Gemini2.0 Flash Thinking.

Figures 3 and 4, along with Supplementary Table 5, present the overall user experience of eight LLMs from a public perspective. Results indicate that Gemini 2.0 Flash Thinking achieved the highest comprehensibility score (4.25, 95% CI 4.14–4.35, *p* < 0.01), while Kimi-K1.5 scored the lowest (3.71, 95% CI 3.59–3.82, *p* < 0.01). Regarding empathy, Gemini models demonstrated superior performance with scores of 3.77 (95% CI 3.67–3.87, *p* < 0.01) and 3.75 (95% CI 3.65–3.85, *p* < 0.01), respectively. DeepSeek-R1 scored the lowest at 3.33 (95% CI 3.19–3.46, *p* < 0.01). In the assessment of perceived value, ChatGPT-4o scored highest (3.87, 95% CI 3.76–3.97, *p* < 0.01), while DeepSeek-R1 scored lowest (3.61, 95% CI 3.51–3.71, *p* < 0.01). Regarding satisfaction performance, Gemini 2.0 Flash achieved the highest average score (3.83, 95% CI 3.76–3.91, *p* < 0.01), while DeepSeek-R1 scored the lowest (3.44, 95% CI 3.34–3.54, *p* < 0.01).

## Discussion

To our knowledge, this study represents the first systematic evaluation of large language models’ ability to comprehend pure-tone audiogram reports. Using a blinded cross-sectional design, we assessed patient-facing hearing diagnosis, interpretation, and personalized recommendation generation capabilities of eight models, which span China and the US, encompass both open-source and closed-source systems, and cover general-purpose and reasoning-oriented large models, across 140 audiogram reports collected from multiple centers. Results indicate that the DeepSeek series models achieved the highest diagnostic accuracy. Expert evaluations demonstrated DeepSeek’s superior professional performance. In multidimensional assessments of patient assistance, multiple models showed potential auxiliary value, with Gemini scoring higher overall than comparable mainstream models like ChatGPT. This study provides a critical baseline for future clinical applications in hearing health.

LLMs still exhibit significant limitations in pure-tone audiometry diagnosis and are highly dependent on the format of input information. Although some models (such as DeepSeek-V3) achieve an accuracy rate of 67% in diagnosing the degree of hearing loss, their performance in diagnosing the nature of hearing loss remains suboptimal (with a maximum accuracy of only 54%). This outcome falls far below LLMs performance in text-based tasks like neurodegenerative diseases^25^, ophthalmology^26^, and radiological lung cancer staging^27^, indicating their limited capability in processing structured chart information and suggesting that relying solely on LLMs for hearing diagnosis lacks clinical reliability. Notably, after introducing supplementary experiments (e.g., providing information combining structured numerical data), the diagnostic accuracy of all six models except DeepSeek significantly improved (*p* < 0.01). We attribute this to potential reasoning errors caused by information overload. This aligns with prior research highlighting current LLMs’ challenges in integrating image and text information, with generally limited accuracy in medical image localization and anomaly detection tasks^28,29^. This demonstrates that LLMs' performance in medical diagnosis depends not only on model capabilities but also closely on task characteristics and input data types.

This study demonstrates that current large language models exhibit a high risk of hallucinations when interpreting audiology reports, potentially misleading diagnostic and intervention recommendations. Among the 100 reports evaluated, Kimi-V1 and ChatGPT-4o showed a hallucination rate of 24%, while Gemini 2.0 Flash Thinking had the lowest rate (4%). This stands in stark contrast to the 0.26% hallucination rate observed in Eric Steimetz et al.‘s study of GPT-4 interpreting pathology reports^30^. These errors often occur systematically in critical areas such as diagnostic inferences and intervention recommendations. For example, diagnostic errors include fabricating hearing threshold values (e.g., incorrectly reporting a pure-tone average) or inventing non-standard hearing classification criteria. Recommendation errors are more common and concerning, such as suggesting non-existent hearing aid models, recommending devices that are technically incompatible with the patient’s audiometric profile (e.g., recommending a device with insufficient gain for a severe-to-profound loss), or proposing inappropriate management pathways. These errors collectively provide users with substantively erroneous and potentially harmful information. Multiple specialty-specific studies (e.g., nursing^31^ and oncology^32,33^) also indicate that while LLMs perform well in primary care settings, they may generate inaccurate content when handling complex specialty issues, underscoring the need for careful evaluation in medical applications.

The readability of LLM outputs is critical for patient comprehension of reports. This study further quantitatively assessed the readability of text outputs from various models, revealing differences in readability when LLMs generate interpretations of hearing reports. Results indicate that only two metrics from the DeepSeek series (FKGL: 6.41, 95%CI 5.93–6.98; FRE: 63.70, 95%CI 59.50–67.30) simultaneously met the National Institutes of Health (NIH) and the American Medical Association (AMA) recommended reading levels for patient materials (FKGL 6-7, FRE 60-70)^34,35^, indicating concise and understandable responses more suitable for non-specialist users. In contrast, Kimi-K1.5’s FKGL corresponds to a high school level (10.17, 95% CI 8.90–11.40), potentially hindering comprehension among users with low health literacy^36^. Regarding response length, Gemini 2.0 Flash produced the longest responses (21,885.54 words, 95% CI 20,864.00–22,899.25), potentially affecting reader patience; DeepSeek-V3 generated the most concise responses (8,131.60 words, 95% CI 7,675.50–8,618.00) without sacrificing key information. Future LLMs must balance information density with readability to avoid compromising professional value through excessive simplification or verbosity.

The results of this study indicate that while LLMs demonstrate potential in interpreting pure-tone audiogram reports, their accuracy remains far below that of clinical practitioners. Expert evaluations reveal that the DeepSeek series models exhibit significant professional advantages. DeepSeek-V3 outperformed R1 in accuracy (3.35, 95% CI 3.14–3.56 and 3.22, 95% CI 3.00–3.43), comprehension and reasoning ability (3.35, 95% CI 3.09-3.61 and 3.24, 95% CI 2.99-3.49), and helpfulness (3.30, 3.08–3.52 and 3.25, 95% CI 3.03–3.47) than other models (*p* < 0.01). Its reports systematically cover key clinical elements such as audiogram interpretation, curve classification, and urgency of intervention, while providing actionable recommendations. In contrast, Gemini 2.0 Flash demonstrated acceptable comprehensiveness (3.14, 95% CI 2.96–3.32) but the lowest accuracy (2.38, 95% CI 2.21–2.55) and reasoning ability (2.38, 95% CI 2.17–2.59), indicating that content richness does not equate to reliable medical judgment. Key issues included misjudging hearing thresholds, failing to correctly map WHO classification criteria, and misinterpreting air-bone gap values, leading to erroneous assessments of the type of hearing loss (e.g., conductive, sensorineural, or mixed). Kimi-V1 and ChatGPT-4o exhibited limitations in comprehensiveness and explanatory depth, consistent with prior research^37^, indicating that current LLMs still face challenges in providing comprehensive and reliable medical explanations.

From the perspective of patient experience, certain models demonstrate unique value in enhancing the patient experience. Notably, unlike clinical experts who rigorously assess LLM performance against professional standards, general users prioritize comprehensibility, linguistic friendliness, psychological comfort in communication, and ease of information access. Research indicates that Gemini 2.0 Flash Thinking achieved the highest score for comprehensibility (4.25, 95% CI 4.14–4.35), while the Gemini series also demonstrated strong empathy scores (3.75, 95% CI 3.65–3.85 and 3.77, 95% CI 3.67–3.87), indicating that its generated content aligns more closely with patients’ communication needs and emotional expectations rather than solely emphasizing medical expertise. While DeepSeek-R1 leads in professional evaluation, it scored lowest in user satisfaction (3.44, 95% CI 3.34–3.54) and empathy (3.33, 95% CI 3.19–3.46), reflecting its high terminology density and inadequate emotional expression, which hinder public comprehension. This highlights patients’ heightened sensitivity to experiential, patient-centered features of AI outputs (e.g., clarity, comprehensibility, and interactivity), which traditional medical explanations often fail to address. However, it should be noted that high comprehensibility does not necessarily equate to reliable medical judgments, suggesting that optimizing a single dimension may lead to imbalances in clinical value. Therefore, we recommend that LLMs applied in hearing health education and communication assistance should balance both the accuracy and reliability of information with the emotional resonance of communication.

This study responds to the World Health Organization’s mission of “hearing health for all^8^.” However, in clinical practice, due to the scarcity of specialized audiology resources and the high workload of physicians in outpatient settings, it is challenging to provide patients with thorough interpretation of examination reports and detailed health recommendations. Compared to traditional hearing reports that only include diagrams and diagnostic conclusions, LLMs can help patients quickly develop a scientific understanding of their own auditory function by converting specialized data into accessible language as a potential auxiliary tool. This approach prevents anxiety caused by delayed interpretation or misunderstanding, while also reducing the risk of “neglecting early intervention due to insufficient awareness.” Especially in regions lacking audiological diagnostic resources, information provided by LLMs is highly likely to become patients’ primary source of medical explanations, partially compensating for insufficient professional communication. However, it must be made clear that patients’ high satisfaction with model outputs should not lead them to equate the results with clinical diagnoses^38,39^. Therefore, it is essential to incorporate prominent risk warnings in system design, emphasizing that the model serves only as an auxiliary interpretation rather than a medical conclusion. Thus, in clinical practice, LLMs may serve more practically as supplementary tools rather than independent decision-makers. When receiving LLM interpretations, patients should clearly understand that these represent only auxiliary opinions, with final diagnoses and treatment decisions relying on professional medical judgment.

There are also some shortcomings in this study. Firstly, data representativeness. This study only included pure-tone audiometry reports from Chinese populations and did not encompass medical histories, speech audiometry, or multimodal data (e.g., ABR and OAE). Additionally, the dataset demonstrated an uneven distribution of hearing loss severities, with mild-to-moderate cases predominating, aligning with population trends where mild and moderate hearing loss are more common than severe or profound cases^4,5,40^. Moreover, this pattern aligns with epidemiological trends observed in the general Chinese population. A meta-analysis review on the prevalence of hearing loss among Chinese seniors indicates that moderate hearing loss (41%) is more prevalent than mild (27%) or severe (10.1%) hearing loss, suggesting that early-stage hearing loss is more common^41^. This imbalance may limit the generalizability of our findings to populations with more severe hearing loss, which are less frequently observed in clinical practice. While this is unlikely to impact the primary comparative analysis, it underscores the need for validation in more representative cohorts.

Second, the manual evaluation may be influenced by human factors. Although all tasks were independently completed by blinded assessors who were unaware of which output corresponded to which LLM, subjective biases from the assessors could still affect the objectivity and consistency of the evaluation results.

Third, the evaluation dimensions were incomplete and did not quantify the model’s actual impact on clinical decision-making (e.g., intervention delays caused by misdiagnosis rates).

Fourth, our analysis was limited to web-based models for consistency; thus, performance may differ from the same LLMs accessed through their native application or other deployment environments. Furthermore, these platforms did not provide precise parameters used, and online applications may utilize shared data for future training, raising questions about their suitability for research settings.

Fifth, the ongoing rapid evolution of LLMs may limit the validity of current findings when newer versions are released.

In summary, this study represents the first evaluation of LLMs’ ability to comprehend specialized medical chart reports, focusing on their potential application in interpreting pure-tone audiograms for patients. It covers model-generated patient-facing hearing diagnoses, explanatory interpretations, and personalized health recommendations. The findings indicate that while existing models, including DeepSeek-V3, still exhibit significantly lower diagnostic accuracy than professional audiologists and produce varying degrees of “hallucination” outputs, rendering them unsuitable for independent clinical diagnosis, they demonstrate clear auxiliary potential and patient value in translating specialized audiograms into patient-understandable report interpretations and health recommendations. This study not only addresses the gap in evaluating LLM applications within audiology but also reveals the critical impact of task characteristics and input formats on LLM medical performance. It highlights the current limitations of LLMs in achieving professional accuracy in chart interpretation while underscoring their potential for generating patient-specific explanations and recommendations. Through optimized prompt engineering, enhanced domain knowledge integration, and the development of human-AI collaborative systems, LLMs hold promise as vital auxiliary tools to bridge global hearing healthcare gaps and mitigate inequitable distribution of medical resources. Their core value lies in improving patient comprehension and health information accessibility, thereby providing essential technological support toward achieving “accessible, inclusive, and precise” universal hearing healthcare^8^.

## Methods

**Fig. 5:**
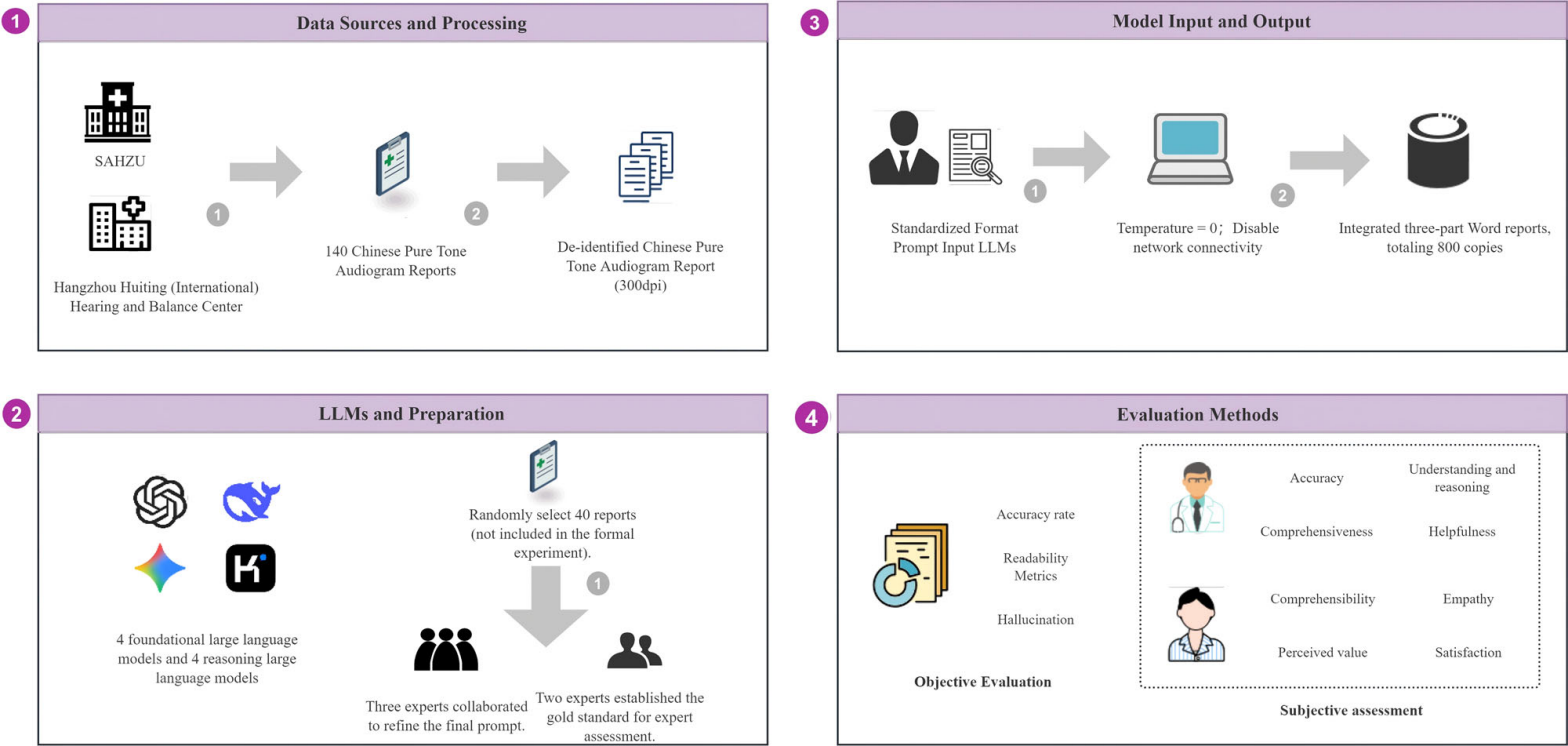
Overall workflow for evaluating pure-tone audiograms based on large language models. (**1**) Data Resources and Processing: Collected 140 pure-tone audiogram reports from two institutions (Second Affiliated Hospital of Zhejiang University SAHZU and Hangzhou Hearing Center), each containing personal information, bilateral audiograms, and clinical diagnoses. For privacy and blind evaluation purposes, de-identified audiograms (300dpi) were regenerated from the original data. (**2**) LLMs and Preparation: Select 8 LLMs from OpenAI, Google, DeepSeek, and Moonshot (4 open-source + 4 closed-source); Forty reports were randomly selected for expert prompt design and gold standard establishment (not included in the formal experiment). (**3**) LLMs Input and Output: Researchers posed three consecutive questions to each model using standardized prompts and sample reports, collecting: **a** Diagnosis (left/right ear “degree + nature,” requiring reasoning process); **b** Interpretation (patient-friendly simplified report); **c** Recommendation (professional opinion). (**4**) Evaluation Methods: Based on the QUEST framework, each model’s responses are evaluated through both objective and subjective assessments.

The workflow of this study is shown in Fig. 5. For the detailed design of the overall framework, please refer to Supplementary Fig. 3. As the evaluation targets a chatbot, the report follows the Chatbot Evaluation Report Tool (CHART) guidelines (BMJ, August 2025)^42^, with the full checklist in Supplementary Table 6. The study protocol was prospectively registered on the Open Science Framework^43^.

### Data resources and processing

The pure-tone audiogram reports for this study were obtained from: the Affiliated Hospital of Zhejiang University School of Medicine (SAHZU) and Hangzhou Huitin (International) Hearing and Balance Center. A total of 140 Chinese pure-tone audiogram reports (including bilateral audiograms and corresponding clinical diagnoses) were randomly collected between October 2024 and December 2024. The specific data collection process and inclusion/exclusion criteria are detailed in Supplementary Note 1. Seventy reports originated from SAHZU, and 70 from Huitin (International) Audiology and Balance Center. Clinical diagnoses in the pure-tone audiogram reports included the degree and nature of bilateral hearing loss. We categorized each ear based on hearing loss severity; the specific sample size distribution is shown in Supplementary Table 7. Based on the original hearing data, we regenerated standardized, anonymized pure-tone audiogram reports without diagnostic outcomes. Specific examples are shown in Supplementary Fig. 4. This study received ethical approval from the SAHZU Faculty of Medicine Ethics Committee(2025–0027). According to the SAHZU Faculty of Medicine Ethics Committee regulations, the use of these anonymized data does not require approval or informed consent. The Institutional Review Board has conditionally approved sharing data access with third parties.

### LLMs selection and preparation

This study selected four latest open-source and closed-source LLMs from four leading companies in China(DeepSeek, Moonshotand) the US (OpenAI, Google). Specific model version dates are listed in Supplementary Table 8.

We randomly sampled 40 reports from the experimental dataset for prompt design and gold standard evaluation. To prevent data leakage, these reports were not reused in subsequent experiments. On one hand, one large model expert (GXW) and two experts with relevant certifications and extensive clinical experience (CHT, MC) conducted multi-round pre-experiments using the 40 cases in Hangzhou, China (January 3–15, 2025). On the other hand, Experts provided prompts and contextual information to the large language model based on existing literature and clinical experience^37,44–49^. After each round, expert feedback was collected, and prompts and experimental design were adjusted accordingly. Specific prompts are detailed in Supplementary Table 9. Separately, two experts (CHT and MC) independently interpreted the 40 reports and provided recommendations, independent of the LLMs outputs, establishing the gold standard for the interpretation and recommendation sections. Additionally, the gold standard for the diagnosis section was the original diagnostic findings.

### LLMs input and output

The collection of input and output results for LLMs was conducted by two trained graduate students (QCF and TTZ) from January 22, 2025, to March 20, 2025. The specific operational procedure was as follows: First, the LLMs generated diagnostic results based on prompts, including assessments of hearing loss severity in both ears and the nature of the hearing loss (e.g., left ear: moderate hearing loss; left ear: sensorineural hearing loss; and the right ear had severe hearing loss with a mixed hearing loss nature). The LLMs were also required to provide the diagnostic reasoning process associated with these diagnoses. Second, the LLMs were tasked with interpreting and providing recommendations based on the pure-tone audiogram report, delivering open-ended explanations and professional advice in a manner accessible to the general public. Additionally, to prevent random responses from the LLMs, each model’s temperature parameter was set to 0, and offline mode was used. This process employed a continuous question-and-answer format, posing three diagnostic, interpretive, and recommendation queries to the LLMs via an online platform. All generated content was collected into structured response reports (100 in total), with specific examples available in Supplementary Table 10. All collected data were ultimately reviewed and verified by the third graduate student (MYX).

### Evaluation methods

We extracted certain subjective metrics from the QUEST framework proposed by Tam et al.^50^ and combined them with the most commonly used objective metrics in LLMs evaluation, including accuracy, readability, and hallucination-related indicators^28,51^, to form an evaluation metric system for diagnosing, interpreting, and recommending LLMs-generated content, as shown in Table 1. To ensure the objectivity of the evaluation, this study employed a blind evaluation design, where the information about the models to be evaluated was concealed from the evaluators to control potential evaluation biases.

**Table 1:** Evaluation Indicators and Standards

	Evaluation indicators	Evaluator	Evaluation criteria	Evaluation results
Objective evaluation	Diagnostic accuracy	1 graduate student in audiology (MYX)	Evaluate only the diagnostic findings section. Compare with the original report’s diagnostic results and record the percentage of correctly diagnosed cases among 200 ears (including accuracy rates for diagnosing hearing loss severity and nature). Additionally, we have incorporated two supplementary tests: a pure-tone audiogram based on raw data (hearing thresholds for each frequency band in both ears) and a combined report featuring both audiograms and tables. (see Supplementary Figs. 5 and 6, 300 dpi format).	Percentage (0–100%)
	Readability-FRE^53^	Web-based tools (readable.com)	Only evaluate the interpretation section, calculated based on sentence length and lexical difficulty. A higher score indicates a shorter text with a simpler structure.	value (1–100)
	Readability-FKGL^54,55^		Only the interpretation section will be evaluated. This scoring assesses the text’s “grade level” based on sentence length and lexical complexity.	Value (0–18)
	Readability-text length		Response length for statistical diagnosis, interpretation, and recommendations	Value (word count)
	Hallucination^56^	2 experts (CHT, MC)	Evaluate the content of the diagnostic process, interpretation, and recommendations. Did the LLMs generate medical findings not present in the original report, or fabricate nonexistent medical data?	Categories (presence or absence)
Subjective assessment	Accuracy^57^	Expert evaluation (2 experts: CHT, MC)	Evaluate the content of the diagnostic process, interpretation, and recommendations. The primary focus is on assessing whether responses are factually correct, precise, and error-free.	5-point Likert scale
	Understanding and reasoning^58^		The evaluation focuses solely on the diagnostic component, primarily assessing whether the large model correctly understands the problem and infers the correct outcome. This includes fundamental comprehension abilities, logical reasoning capabilities, and clinical diagnostic support.	5-point Likert scale
	Comprehensive^59^		Evaluate only the interpretation and recommendation sections. The primary focus is on assessing the completeness of the responses provided by the LLMs. Does the response cover all key aspects, offering a comprehensive overview or detailed insights?	5-point Likert scale
	Helpfulness^60^		Evaluate only the recommendation section. This refers to the applicability and practicality of the response. Determine whether the answer holds real-world value and whether it is actionable and relevant to the user’s question.	5-point Likert scale
	Comprehensibility/understandability^61^	Public evaluation (3 volunteers without medical backgrounds: MJL, YF, and YCQ)	The ease with which users can comprehend the information and content provided by the LLMs.	5-point Likert scale
	Empathy^62^		The ability of an LLMs to empathize with others’ emotions, thoughts, and circumstances, and to respond appropriately with emotional sensitivity.	5-point Likert scale
	Perceived value/Usefulness^63^		Does the information or content provided by the LLMs genuinely prioritize the user’s perspective, address their core needs, and deliver tangible benefits?	5-point Likert scale
	Satisfaction^64^		Users’ subjective perceptions and level of recognition regarding the overall output quality and practicality of LLMs.	5-point Likert scale

### Statistical analysis

Statistical analyses were performed using IBM SPSS Statistics version 29.0.1.0^52^. Descriptive statistics are reported as mean ± standard deviation for continuous variables, with confidence intervals calculated using Wilson’s scoring method. For the continuous readability metrics, the Friedman test was employed as a robust alternative after confirming that the normality assumption for parametric repeated-measures ANOVA was violated. Since Likert scale scores are ordinal and do not meet the normality assumption required for parametric tests, the Friedman test was used to compare the overall performance of different large language models, supplemented by Nemenyi post-hoc tests. Mann-Whitney U tests were applied for independent sample comparisons. Expert assessment consistency was analyzed using Cohen’s Kappa. *P* < 0.05 were considered statistically significant.

## Supplementary information

Supplementary information

## Data Availability

Due to the sensitive nature of the data (e.g., patient information), access is restricted. Data are available from the corresponding author upon reasonable request and with permission from the Human Research Ethics Committee, Second Affiliated Hospital, School of Medicine, Zhejiang University.
