# Supplementary material for: A multicenter multifunctional assessment of large language models in pure-tone audiogram interpretation for patients
Source: NPJ Digit Med. 2026 Mar 15;9:348. doi: 10.1038/s41746-026-02537-1 (PMC13136335; doi:10.1038/s41746-026-02537-1)
Supplement: Supplementary file 1 — Supplementary information [file 41746_2026_2537_MOESM1_ESM.pdf]

Supplementary Information

For Manuscript:

“A Multicenter Multifunctional Assessment of Large Language Models in Pure-Tone Audiogram Interpretation for Patients”

Contents

Supplementary Figures 1-6 2

Supplementary Tables 1-10 8

Supplementary Note 1 30

Supplementary References 31

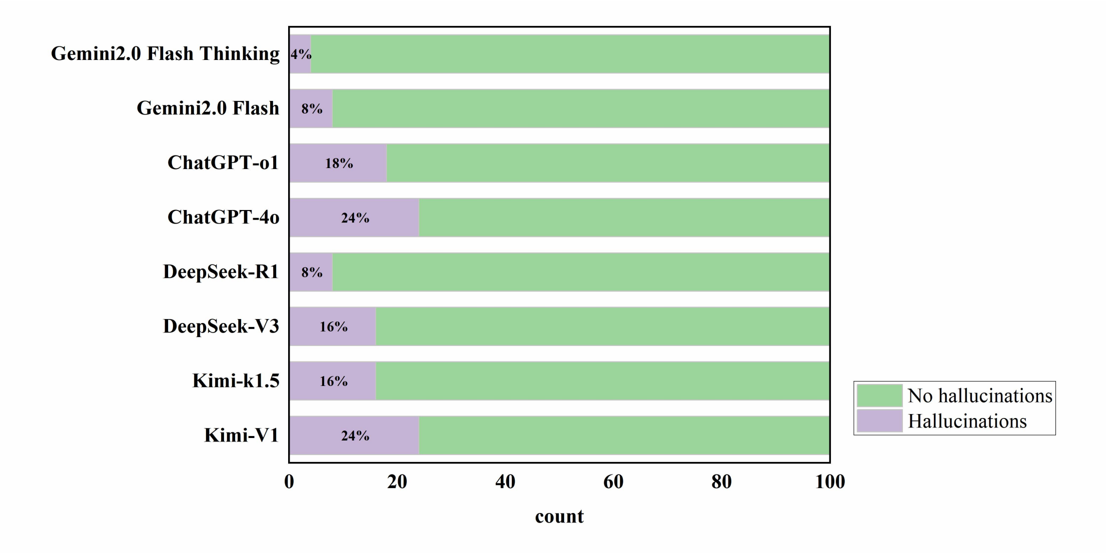

Supplementary Figure 1. Distribution of hallucinations in various large language models. The bar chart shows the percentage of hallucinations (purple) and non-hallucinations (green) across different models, including Gemini2.0 Flash Thinking, Gemini2.0 Flash, ChatGPT-01, ChatGPT-4o, DeepSeek-R1, DeepSeek-V3, Kimi-k1.5, and Kimi-V1.

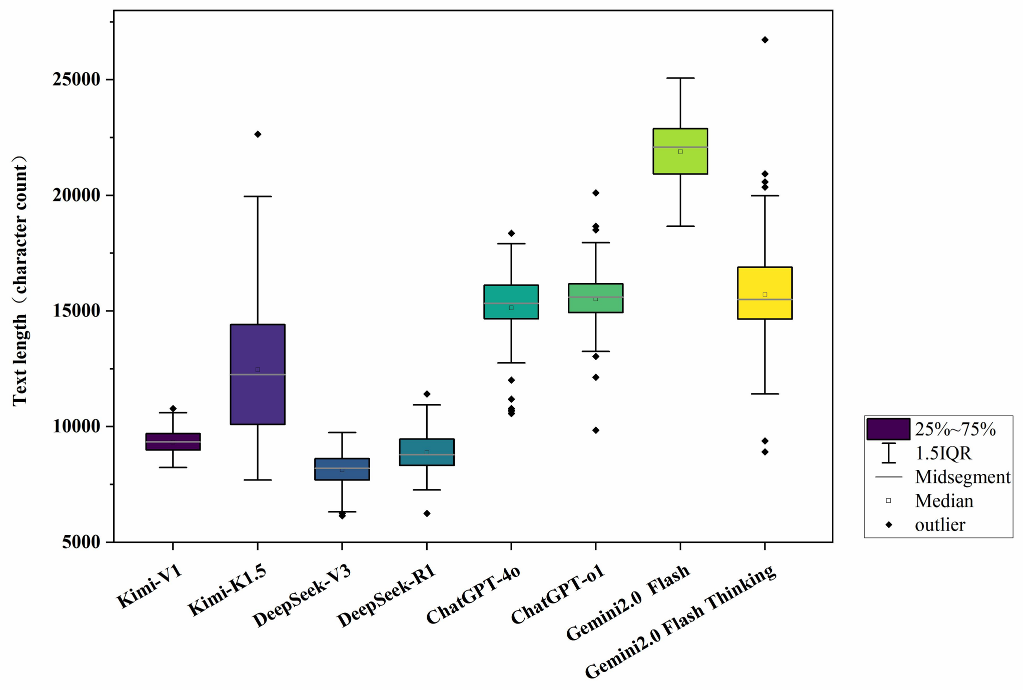

Supplementary Figure 2. Distribution of text length (character count) across different LLMs. The box plot illustrates the median, interquartile range (IQR), and outliers for the text length produced by various models, including Kimi-V1, Kimi-K1.5, DeepSeek-R1, DeepSeek-V3, ChatGPT-4o, ChatGPT-01, Gemini2.0 Flash, and Gemini2.0 Flash Thinking. The box plot illustrates the median, interquartile range (IQR), and outliers for the text length produced by various models, including Kimi-V1, Kimi-K1.5, DeepSeek-R1, DeepSeek-V3, ChatGPT-4o, ChatGPT-01, Gemini2.0 Flash, and Gemini2.0 Flash Thinking.

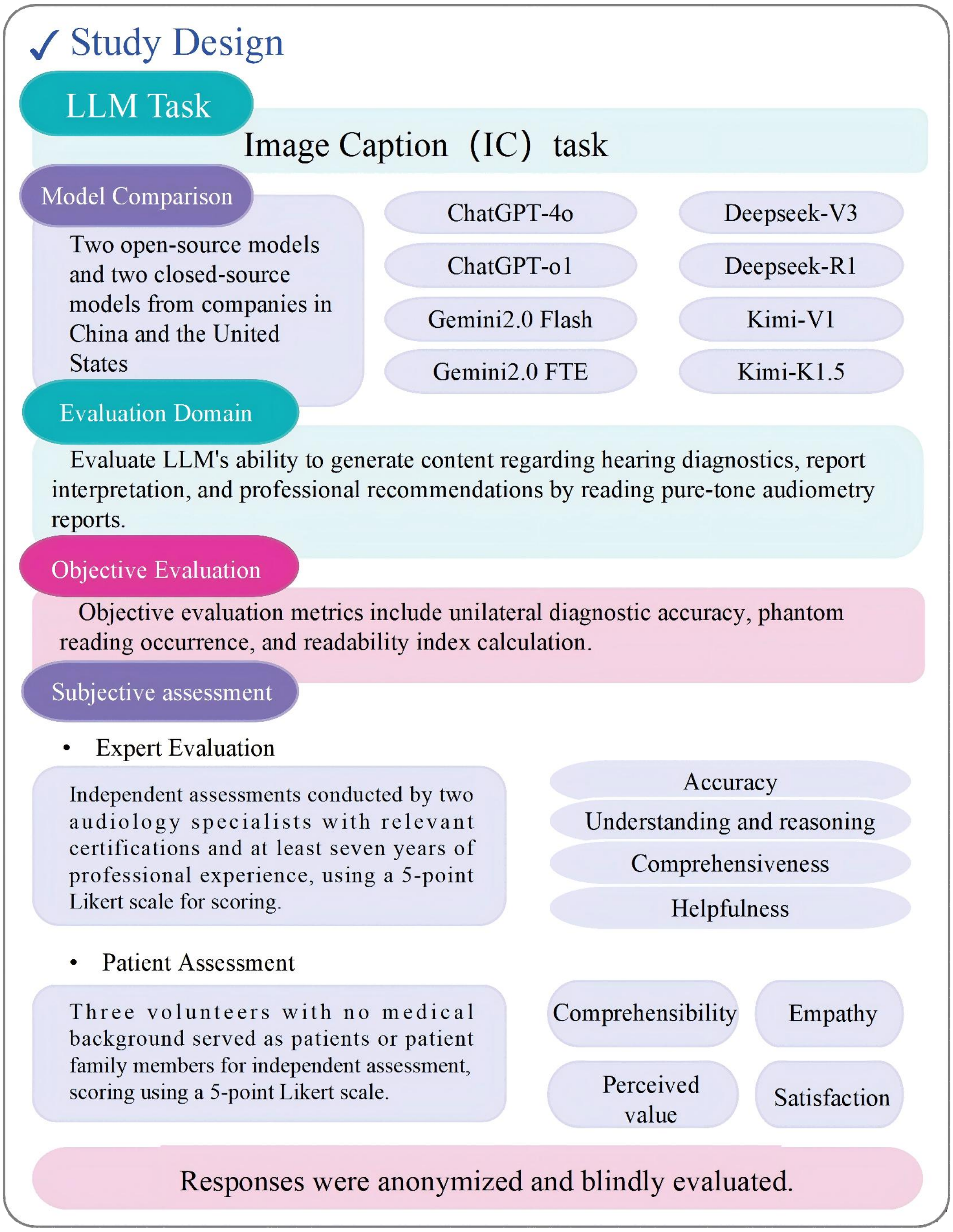

Supplementary Figure 3. Study Design. This diagram outlines the model comparison between open-source and closed-source models from companies in China and the United States, the evaluation domain focusing on hearing diagnostics and audiogram interpretation, and the objective and subjective assessment methods. The objective evaluation includes diagnostic accuracy and readability index, while the subjective assessment incorporates expert and patient evaluations based on a 5-point Likert scale, focusing on various criteria like accuracy, understanding, comprehensibility, and empathy.

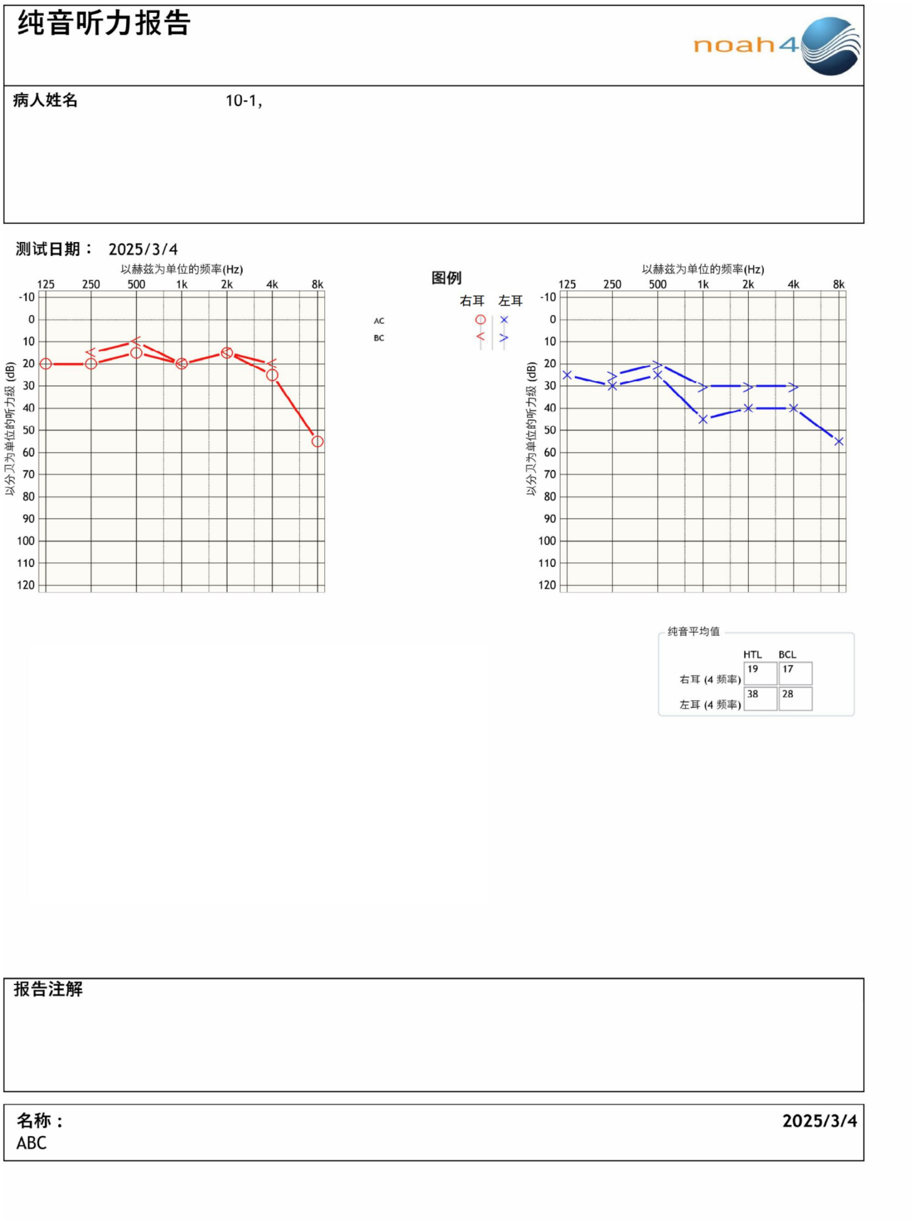

Supplementary Figure 4. Example of an anonymized pure-tone audiogram report generated from raw data. This report displays hearing thresholds (AC and BC) for different frequencies, with annotations for air conduction (AC) and bone conduction (BC) thresholds. The red line represents the right ear (AC: ○; BC: <), while the blue line represents the left ear (AC: ×; BC: >).The audiogram also includes sample case data, with hearing threshold levels (HTL) and bone conduction levels (BCL) for both ears.

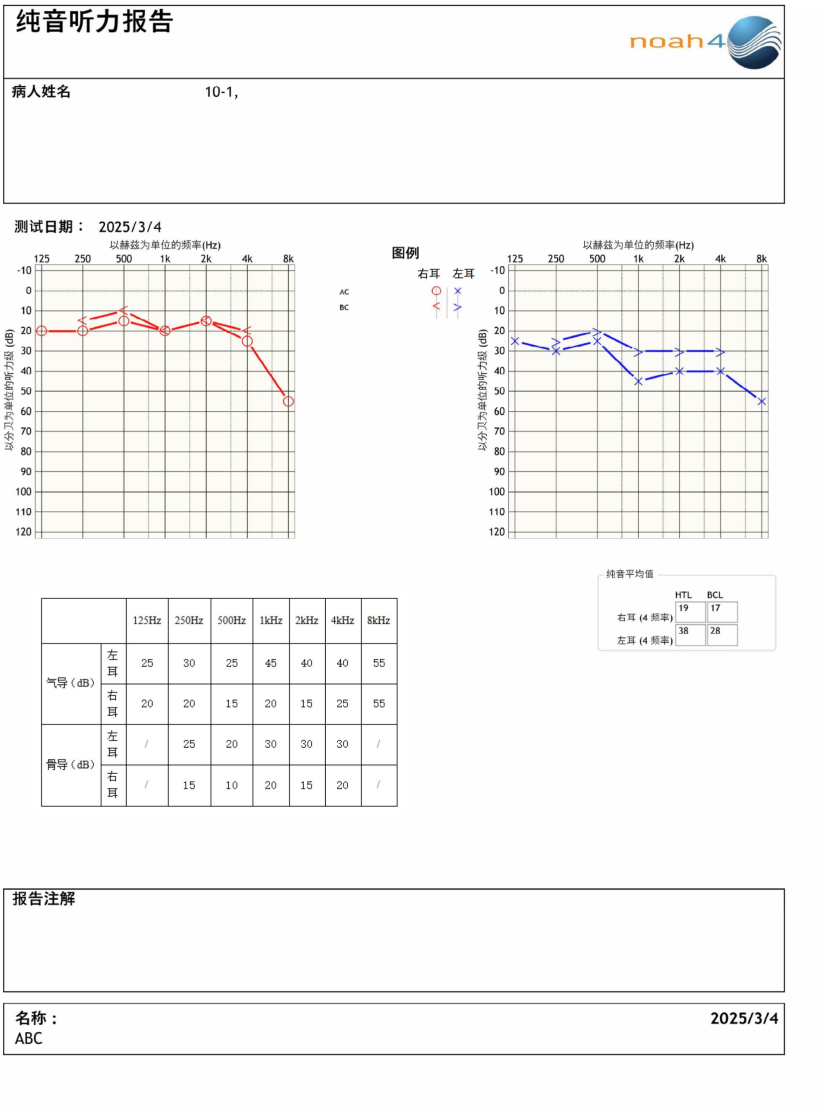

纯音听力报告

noah4

病人姓名 10-1,

测试日期： 2025/3/4

图例

	右耳	左耳
AC	○	×
BC	<	>

纯音平均值

	HTL	BCL
右耳 (4 频率)	19	17
左耳 (4 频率)	38	28

		125Hz	250Hz	500Hz	1kHz	2kHz	4kHz	8kHz
气导（dB）	左耳	25	30	25	45	40	40	55
	右耳	20	20	15	20	15	25	55
骨导（dB）	左耳	/	25	20	30	30	30	/
	右耳	/	15	10	20	15	20	/

报告注解

名称： 2025/3/4
ABC

Supplementary Figure 5. Example of a Pure-Tone Audiogram Report with Detailed Hearing Thresholds. This report displays hearing thresholds (AC and BC) for different frequencies, with annotations for air conduction (AC) and bone conduction (BC) thresholds. The red line represents the right ear (AC: ○; BC: <), while the blue line represents the left ear (AC: ×; BC: >). The table below provides the specific air conduction and bone conduction threshold values (in dB) for both ears at various frequencies. The audiogram also includes hearing threshold levels (HTL) and bone conduction levels (BCL) for both ears.

		125Hz	250Hz	500Hz	1kHz	2kHz	4kHz	8kHz
气导（dB）	左耳	25	30	25	45	40	40	55
	右耳	20	20	15	20	15	25	55
骨导（dB）	左耳	/	25	20	30	30	30	/
	右耳	/	15	10	20	15	20	/

Supplementary Figure 6. Table image of Hearing Thresholds Extracted from the Audiogram Report. This table includes the detailed hearing thresholds from the original pure-tone audiogram, presenting the air conduction (AC) and bone conduction (BC) threshold values (in dB) for both the right ear and the left ear at various frequencies (125 Hz, 250 Hz, 500 Hz, 1 kHz, 2 kHz, 4 kHz, and 8 kHz).

Supplementary Table 1. Accuracy Performance of LLM in Hearing Report Diagnosis

	Official Experiment		Supplementary Experiment 1 - Pure-Tone Audiogram Report with Detailed Hearing Thresholds.				Supplementary Experiment 2 -Table image of Hearing Thresholds Extracted from the Audiogram Report.			
	Degree of Hearing Loss	Nature of Hearing Loss	Degree of Hearing Loss	P value	Nature of Hearing Loss	P value	Degree of Hearing Loss	P value	Nature of Hearing Loss	P value
Kimi-V1	0.36	0.40	0.54	<0.01	0.46	<0.01	0.69	<0.01	0.49	<0.01
Kimi-K1.5	0.55	0.43	0.63	<0.01	0.59	<0.01	0.87	<0.01	0.58	<0.01
DeepSeek-V3	0.67	0.54	0.31	1	0.37	<0.01	0.32	1	0.36	<0.01
DeepSeek-R1	0.65	0.47	0.32	0.514	0.36	<0.01	0.32	0.514	0.35	<0.01
ChatGPT-4o	0.53	0.35	0.51	<0.01	0.38	<0.01	0.73	<0.01	0.49	<0.01
ChatGPT-o1	0.44	0.36	0.48	<0.01	0.40	<0.01	0.71	<0.01	0.46	<0.01
Gemini 2.0 Flash	0.34	0.32	0.49	<0.01	0.46	<0.01	0.94	<0.01	0.63	<0.01
Gemini 2.0 Flash Thinking	0.24	0.42	0.44	<0.01	0.46	<0.01	0.95	<0.01	0.60	<0.01

*Significance p-values are compared with the formal trial of the same model.

Supplementary Table 2. Readability Analysis: Statistics on average characters per LLM response, FKGL, and FRE

	Kimi V1	Kimi K1.5	DeepSeek V3	DeepSeek R1	ChatGPT 4o	ChatGPT o1	Gemini2.0 Flash	Gemini2.0 Flash Thinking	P value
**Character count, Mean(IQR)**	9327.25 (8986.25-9697.25)	12462.72 (10088.25-14423.25)	8131.60 (7675.50-8618.00)	8872.36 (8311.75-9467.50)	15139.95 (14655.50-16125.75)	15523.41 (14904.25-16192.50)	21885.54 (20864.00-22899.25)	15705.48 (14641.75-16906.00)	<0.01
**FKGL, Mean(IQR)**	7.53 (7.10-7.90)	10.17 (8.90-11.40)	6.83 (6.40-7.30)	6.41 (5.93-6.98)	9.50 (9.20-10.10)	9.68 (9.30-10.10)	9.26 (9.00-9.60)	8.57 (8.03-9.08)	<0.01
**FRE, Mean (IQR)**	66.05 (63.08-68.90)	44.71 (38.90-50.50)	61.48 (58.23-64.85)	63.70 (59.50-67.30)	53.59 (51.03-55.85)	53.20 (50.63-55.18)	59.96 (57.83-61.88)	65.30 (62.65-67.55)	<0.01

Supplementary Table 3. Expert evaluation of LLM performance in diagnosing, interpreting, and providing recommendations for hearing reports

	Kimi V1	Kimi K1.5	DeepSeek V3	DeepSeek R1	ChatGPT 4o	ChatGPT o1	Gemini2.0 Flash	Gemini2.0 Flash Thinking	P value
**Accuracy**	2.51（2.30-2.72）	2.99（2.74-3.24）	3.35（3.14-3.56）	3.22（3.00-3.43）	2.74（2.50-2.98）	2.66（2.42-2.90）	2.38（2.21-2.55）	2.45（2.26-2.64）	<0.01
**Understanding and reasoning**	2.57（2.32-2.82）	2.97（2.69-3.25）	3.35（3.09-3.61）	3.24（2.99-3.49）	2.80（2.53-3.07）	2.66（2.41-2.91）	2.38（2.17-2.59）	2.55（2.33-2.77）	<0.01
**Comprehensiveness**	2.45（2.29-2.61）	2.89（2.69-3.09）	3.19（2.99-3.39）	3.08（2.91-3.25）	2.95（2.76-3.14）	2.82（2.63-3.00）	3.14（2.96-3.32）	2.88（2.71-3.05）	<0.01
**Helpfulness**	2.69（2.51-2.87）	3.17（2.94-3.40）	3.30（3.08-3.52）	3.25（3.03-3.47）	3.07（2.84-3.30）	2.95（2.72-3.18）	2.80（2.61-3.00）	2.77（2.57-2.97）	<0.01

Supplementary Table 4. LLMs' performance in comprehensibility, empathy, perceived value, and satisfaction when interpreting hearing reports

	Kimi V1	Kimi K1.5	DeepSeek V3	DeepSeek R1	ChatGPT 4o	ChatGPT o1	Gemini2.0 Flash	Gemini2.0 Flash Thinking	P value
**Comprehensibility**	4.09（3.99-4.18）	3.71（3.59-3.82）	3.97（3.86-4.07）	3.77（3.67-3.87）	4.13（4.03-4.22）	4.14（4.03-4.25）	4.14（4.06-4.22）	4.25（4.14-4.35）	<0.01
**Empathy**	3.49（3.40-3.58）	3.33（3.23-3.43）	3.42（3.31-3.52）	3.33（3.19-3.46）	3.48（3.37-3.59）	3.34（3.23-3.45）	3.77（3.67-3.87）	3.75（3.65-3.85）	<0.01
**Perceived value**	3.66（3.56-3.77）	3.74（3.65-3.83）	3.62（3.53-3.71）	3.61（3.51-3.71）	3.87（3.76-3.97）	3.66（3.56-3.76）	3.77（3.69-3.85）	3.67（3.58-3.75）	<0.01
**Satisfaction**	3.66（3.58-3.76）	3.53（3.43-3.62）	3.63（3.53-3.73）	3.44（3.34-3.54）	3.76（3.67-3.85）	3.64（3.54-3.73）	3.83（3.76-3.91）	3.71（3.63-3.79）	<0.01

Supplementary Table 5. Results of expert assessment consistency

Indicator	Cohens Kappa (95% CI)	P value
Accuracy	0.89 (0.83-0.95)	<0.001
Understanding and Reasoning ability	0.82 (0.79-0.85)	<0.001
Helpfulness	0.72 (0.65-0.79)	<0.001
Comprehensive	0.71(0.65-0.78)	<0.001

Supplementary Table 6. CHART Checklist for Research Methods and Reporting Srandards in LLM Evaluation[1]

Heading	Item No	CHART CHECKLIST ITEM	CHECJLIST ITEM FOR THIS STUDY	Page No
**Title & Abstract**				
**Title**	**1a**	State that the study is assessing one or more generative AI-driven chatbots for clinical evidence or health advice.	Title Statement: Multiple generative AI large models are being evaluated for the diagnosis, interpretation, and recommendations of pure-tone audiometry reports in the field of audiology.	1
**Abstract/Summary**	**1b**	Apply a structured format, if applicable.	Structured Format Abstract	3-4
**Introduction**				
**Background**	**2a**	State the scientific background, rationale, and healthcare context for evaluating the generative AI-driven chatbot(s), referencing relevant literature when applicable.	Provide background information on the scientific context, hearing healthcare context, and societal needs.	4
	**2b**	State the aims and research questions including the target audience, intervention, comparator(s), and outcome(s).	The introduction clearly states the research objectives and research questions of this study.	5
**Methods**				
**Model Identifiers**	**3a**	State the name and version identifier(s) of the generative AI model(s) and chatbot(s) under evaluation, as well as their date of release or last update.	The method specifically lists each LLM's name, affiliated company, version identifier, and exact release date.	20,Supplementary Table8
	**3b**	State whether the generative AI model(s) and chatbot(s) are open-source or	Specify whether the specific LLM is open-source or closed-source.	20,Supplementary Table8

		closed-source/proprietary.		
**Model Details**	**4a**	State whether the generative AI model was a base model or a novel base model, tuned model, or fine-tuned model.	Description: a novel base model	Supplementary Table 8
	**4b**	If a base model is used, cite its development in sufficient detail to identify the model.	The article explains the parameters when using LLM.	21, Supplementary Table 8
	**4c**	If a novel base model, tuned model, or fine-tuned model is used, describe the pre- and/or post-implementation/deployment data and parameters.	The method details the specific configuration and parameters of the LLM.	21
**Prompt Engineering**	**5a**	Describe the evolution of study prompt development.	Description Prompt Development Process	20
	**5ai**	Describe the sources of prompts.	Describe the source of prompts in the evaluation process	20
	**5aii**	State the number and characteristics of the individual(s) involved in prompt engineering.	Describe the number and characteristics of experts participating in the prompt engineering project.	20
	**5aiii**	Provide details of any patient and public involvement during prompt engineering.	Briefly describes the information involved in the prompt engineering project.	20
	**5b**	Provide study prompts.	We have provided the complete prompt in the appendix.	Supplementary Table 9
**Query Strategy**	**6a**	State route of access to generative AI model.	Specifies the access path for the LLM	21
	**6b**	State the date(s) and location(s) of queries for the generative AI-driven chatbot(s) including the day, month, and year as well as city and	The query period is from January 22, 2025, to March 20, 2025, with the location in Zhejiang Province, China.	20, 21

		country.		
	**6c**	Describe whether prompts were input into separate chat session(s).	Provide specific ways to ask questions of LLM models (ask each model three questions for diagnosis, interpretation, and recommendations; each audiogram case will generate a separate dialogue box).	21
	**6d**	Provide all generative AI-driven chatbot output/responses.	Due to patient privacy concerns, only partial outputs from the LLM will be provided.	Supplementary Table 10
**Performance Evaluation**	**7a**	Define the ground truth or reference standard used to define successful generative AI-driven chatbot performance.	Describe specific evaluation metrics and scoring criteria.	21, 22, Figure 1, Table 1, Supplementary Figure 1
	**7b**	Describe the process undertaken for generative AI-driven chatbot performance evaluation.	Describe the specific process of evaluating large language models (LLMs)	21, 22, Figure 1
	**7bi**	State the number and characteristics of team members involved in performance evaluation.	Specify the number of participants in the evaluation and their specific characteristics.	22, Table 1
	**7bii**	Provide details of any patients and public involvement during the evaluation process.	Privacy concerns prevent disclosure at this time.	
	**7biii**	State whether evaluators were masked to the identity of the generative AI-driven chatbot(s) under assessment.	The evaluation was conducted using a blind method (with LLM identifiers removed).	22
**Sample Size**	**8**	Report how the sample size was determined.	Describe the sample size, collection sites, time periods, and inclusion criteria.	19, 20, Supplementary Note1

**Data Analysis**	**9a**	Describe statistical analysis methods, including any evaluation of reproducibility of generative AI-driven chatbot responses.	In the methodology section, descriptive statistical analysis methods are described, including significance analysis for each indicator and consistency analysis of expert evaluations.	22
	**9ai**	Report the measures used for performance evaluation.	Description of measurement methods used for performance evaluation (readability)	Table 1
**Results**				
	**10a**	Report the alignment between generative AI-driven chatbot output and ground truth or reference standard using quantitative or mixed methods approaches as applicable.	Use scientific quantitative methods to report the alignment between LLM outputs and true values or reference standards.	6-10
	**10b**	For responses deviating from the ground truth or reference standard, state the nature of the difference(s).	Expand the explanation in the article results section.	6-10
	**10c**	Report the assessment for potentially harmful, biased, or misleading responses.	Illustrating instances where LLM responses exhibit hallucinations, fabrications, and generate misleading replies through examples.	6, Supplementary Figure 1
**Discussion**				
	**11a**	Interpret study findings in the context of relevant evidence.	Interpret the findings in light of relevant evidence, including prior research.	10-18
	**11b**	Describe the strengths and limitations of the study.	Describe the strengths and limitations of the study in the discussion.	16, 17
	**11c**	Describe the potential implications for practice, education, policy, regulation, and research.	Describe the significance of the research and propose the potential application prospects of LLM in the field of hearing.	18
**Open Science**				

**Disclosures**	**12a**	Report any relevant conflicts of interest for all authors.	All relevant conflicts of interest for the authors will be reported at the end of the article.	24
**Funding**	**12b**	Report sources of funding and their role in the conduct and reporting of the study.	Funding support will be acknowledged in the acknowledgments section at the end of the article.	23
**Ethics**	**12c**	Describe the process undertaken for ethical approval.	Provide documentation of ethical review approval	Related documents
	**12ci**	Describe the measures taken to safeguard data privacy of patient health information, as applicable.	The Methods section describes the measures taken to remove patient privacy.	19, 20
	**12cii**	State whether permission/licensing was obtained for the use of original, copyrighted data.	Permission to access the original copyright data has been granted following ethical review.	19, 20, Related documents
**Protocol**	**12d**	Provide a study protocol.	The relevant research proposals are presented in the appendix section.	18,Supplementary Figure 3
**Data availability**	**12e**	State where study data, code repository, and model parameters can be accessed.	Data availability is reported at the end of the article.	23

Supplementary Table 7. Sample Size Distribution for Different Degrees of Hearing Loss Across Multiple Centers

	The Second Affiliated Hospital of Zhejiang University（SAHZU）	HuiTing (International) Hearing and Balance Center
Normal	9	7
Mild hearing loss	24	24
Moderate hearing loss	43	39
Moderately severe hearing loss	32	29
Severe	19	24
Profound hearing loss	8	11
Complete deafness	5	6
Total	140	140

*Note: A report includes the results for both ears.

Supplementary Table 8. Specific details of the selected LLM

Open-source/Closed-source	Parent Company	Version Identifier	Version Date	Model Type
Closed-source Large Model	OpenAI	ChatGPT-4o	version May 13, 2024	Foundation Model
		ChatGPT-o1	version Dec 5, 2024	Reasoning Model
	Google Inc	Gemini 2.0 Flash	versions Feb 5, 2025	Foundation Model
		Gemini 2.0 Flash Thinking	versions Feb 5, 2025	Reasoning Model
Open-source Large Model	DeepSeek	DeepSeek-V3	Dec 26,2024	Foundation Model
		DeepSeek-R1	Jan 20,2025	Reasoning Model
	Moonshot AI	Kimi-V1	version Jan 15,2025	Foundation Model
		Kimi-K1.5	version Jan 20,2025	Reasoning Model

Supplementary Table 9.The prompt used in this study

Prompt strategy	Input
Report of Diagnosis	As an experienced audiologist, you are asked to professionally evaluate the patient's hearing status based on the results of the pure tone hearing test provided by the patient. Please analyze the report step-by-step based on the specific data in the report and follow the chain of thought steps below for detailed reasoning:<br>1. Hearing curve analysis:<br>Please carefully analyze the hearing curves of the patient's pure tone air conduction and bone conduction, pay attention to the overlap of the curves, and identify the patient's hearing data at four frequency points: 0.5 kHz, 1 kHz, 2 kHz and 4 kHz.<br>2. Degree of hearing loss determination:<br>The degree of hearing loss was determined based on the patient's air-conducted mean hearing loss at the four frequency points of 0.5 kHz, 1 kHz, 2 kHz, and 4 kHz, according to the 2021 World Health Organization (WHO) Hearing Loss Grading Criteria. The specific grading criteria are as follows:<br>- Normal: mean hearing threshold less than or equal to 20 dB;<br>- Mild hearing loss: mean hearing thresholds of 21-34 dB;<br>- Moderate hearing loss: mean hearing threshold 35-49 dB;<br>- Moderately hearing loss: average hearing thresholds of 50-64 dB;<br>- Severe hearing loss: average hearing thresholds of 65-79 dB;<br>- Profound hearing loss: average hearing threshold of 80-94 dB;<br>- Complete deafness: average hearing thresholds greater than or equal to 95 dB.<br>The patient's hearing condition may be: (A) Normal, (B) Mild hearing loss, (C) Moderate hearing loss, (D) Moderately severe hearing loss, (E) Severe, (F) Profound hearing loss, (G) Complete deafness.<br>3. Determination of the nature of hearing loss:<br>The nature of the hearing loss is determined based on the patient's air-conduction and bone-conduction hearing in the audiogram. The specific classifications are as follows:<br>- Normal: air-conduction thresholds less than 20 dB.<br>- Conductive Deafness: This is characterized by air-conduction thresholds greater than or equal to 20 dB and bone-conduction thresholds less than or equal to 10 dB, and the air-bone-conduction difference is usually greater than or equal to 10 dB.<br>- Sensorineural Deafness: manifested by air-conduction thresholds greater than or equal to 20 dB and bone-conduction thresholds greater than or equal to 10 dB, and the air-bone-conduction difference is usually less than or equal to 10 dB.<br>- Mixed Deafness: manifested by air-conduction thresholds greater than or equal to 20 dB and bone-conduction thresholds greater than or equal to 10 dB, and the air-bone conductance difference is usually greater than or equal to 10 dB.<br>Determine the nature of the patient's hearing loss, which may be one of the following four properties of hearing loss: (A) Normal, (B) Conductive deafness,

	(C) Sensorineural deafness, and (D) Mixed deafness.<br><br>To complete this task, you will need to analyze each description in the report in depth, using medical knowledge and scientific facts to guide the reasoning process. During your analysis, be sure to explain each step of the reasoning process in detail to make it easier for the patient to understand how you arrived at your conclusions. While it may sometimes be difficult to arrive at a definitive diagnosis based on the report alone, you should also choose the most likely scenario of hearing loss rather than saying it is undiagnosable. Finally, be sure to find as much reliable evidence as possible to support your reasoning process. In your response, follow this structure: "Based on the patient's pure tone hearing report, let's think step-by-step: {Process of reasoning}. Based on our thorough analysis, we can conclude that the degree of hearing in the patient's right ear is {degree of hearing loss option} and the nature of the hearing loss in the right ear is {nature of hearing loss option}, and the degree of hearing in the patient's left ear is {degree of hearing loss option} and the nature of the hearing loss in the left ear is {nature of hearing loss option}."
Report Interpretation	As an experienced audiologist, you are tasked with interpreting a patient's pure tone hearing report, which contains my hearing thresholds, frequency response curves, and hearing loss data in various frequency bands. Interpreting in detail to a patient with only a high school education level, please use concise, clear statements, remain patient, and explain in simple, easy-to-understand language to ensure the patient feels comfortable and understood. Please analyze the report step-by-step based on the specific data in the report and follow the chain of thought steps below for detailed reasoning:<br><br>1. Extract and understand the key information in the report:<br>Please begin by identifying the key data points in the report, including the extent of hearing loss in each frequency band; are there any unusual hearing data or obvious trends? Are there losses in specific frequency ranges?<br><br>2.Analyze the hearing loss in the frequency bands:<br>For each frequency point, please determine if hearing loss exists and identify the following areas:<br>Low frequency band (250Hz-500Hz): Is the low frequency loss normal and could it be caused by age, fluid in the ear canal, etc.?

	Mid-frequency band (1000Hz-2000Hz): is there a prominent hearing loss that may be associated with loss of speech comprehension?<br>High-frequency band (4000Hz-8000Hz): high-frequency loss is usually associated with noise exposure, aging, and other factors. Please analyze the extent of hearing loss in the high frequency bands and assess whether it may be noise-induced.<br>Summarize the differences between the frequency bands. Is there a particular frequency band where the loss is most significant?<br><br>3. Analyze the auditory conduction pathways:<br>Interpret in detail the hearing loss in the air conduction, the hearing loss in the bone conduction, explain whether the pathways in the air conduction and bone conduction are normal, and what parts of the body may be diseased?<br><br>4. Speculate on possible causes of hearing loss:<br>Noise exposure: if high-frequency loss is more significant, consider whether there is a history of long-term noise exposure.<br>Genetic factors: whether any family history is reported or if certain frequency band losses are hereditary.<br>Age factors: aging may contribute to overall hearing loss, especially in the high frequency bands.<br>Health issues: Are there other possible causes (e.g., ear infections, medication side effects, otosclerosis, etc.)?<br>Acute injuries: Has there been any ear trauma or acute noise exposure event?<br><br>5. Assess the severity of the hearing loss:<br>Based on the reported hearing thresholds, please determine the severity of the hearing loss and how the current condition of the patient's hearing loss may result in daily performance in quiet and noisy environments?<br><br>6.Does this hearing report indicate any unusual health problems that require further medical evaluation or treatment recommendations?<br><br>Please follow the above steps of the chain of thought for detailed analysis, step-by-step reasoning and conclusions, and finally provide comprehensive hearing interpretations and recommendations.
Professional advice	As an experienced audiologist, your task is to analyze and give a detailed hearing loss assessment and recommendation based on step-by-step thought reasoning from the patient's pure tone hearing report data. Please follow the thought chain steps below for detailed reasoning:<br>1. Further medical diagnosis<br>Based on the analysis results, determine whether further examination or treatment is required. If the report shows abnormal hearing loss conditions or

	data, please reason out the specific items that require further diagnosis (e.g. speech hearing test, otological examination, etc.). Based on the results of the reasoning, recommend whether more in-depth examination or monitoring is needed to more accurately determine the source and type of hearing loss.<br><br>2. Recommendation of treatment and intervention programs<br>Based on the reasoned type and degree of hearing loss, provide targeted treatment recommendations. Based on the type and degree of loss, provide appropriate treatment programs:<br>Conductive loss: medication or surgery may be required (e.g. ear infection, blockage of ear canal, etc.).<br>Sensorineural loss: devices such as hearing aids and cochlear implants may be considered.<br>Mixed loss: may require a combination of medication, surgery, and hearing assistive devices.<br>Reason about the effectiveness of different treatment modalities and give the appropriate treatment options for the patient.<br><br>3. Lifestyle and Preventive Measures Recommendations<br>Depending on the type and cause of hearing loss, are lifestyle modifications (e.g., hearing protection, avoidance of noise exposure, regular checkups) recommended?<br>Provide preventive measures to help reduce the risk of future hearing loss. Please reason and provide specific preventive and hearing improvement measures:<br>Noise protection: If noise exposure is a cause, it is recommended to wear earplugs or earmuffs to avoid prolonged exposure to high noise levels.<br>Healthy habits: e.g. avoid prolonged use of headphones and frequent listening to loud music.<br>Regular checkups: Regular hearing checkups are recommended, especially for the middle-aged and elderly population.<br>To help minimize the risk of future hearing loss, e.g. use of earplugs, avoidance of long-term ototoxic medications. If genetic or aging factors are involved, is regular monitoring and evaluation necessary?<br><br>Please follow the above chain of thought steps for detailed analysis, step-by-step reasoning and conclusions, and finally provide professional hearing advice.

Supplementary Table 10. Example of a table formed by integrating text content from LLMs outputs

Prompt strategy	Output
Report of Diagnosis	Based on the patient's pure tone hearing report, let's think step-by-step:<br><br>**Hearing Curve Analysis**<br><br>1. From the report's audiogram, we note that both **air-conduction (AC)** and **bone-conduction (BC)** thresholds are elevated for the right and left ears, though to differing extents.<br>2. At the four key frequencies (0.5 kHz, 1 kHz, 2 kHz, and 4 kHz), the **average AC thresholds** (HTL) are reported in the summary as:<br>1. Right ear: 65 dB<br>2. Left ear: 52 dB<br>3. The **average BC thresholds** (BCL) given are:<br><br>1. Right ear: 54 dB<br>2. Left ear: 40 dB<br><br>**Degree of Hearing Loss Determination**<br>Using the 2021 WHO grading (four-frequency average of 0.5, 1, 2, and 4 kHz):<br><br>4. **Right Ear (AC average = 65 dB)**<br><br>1. According to WHO: 65–79 dB average = **Severe hearing loss**.<br><br>5. **Left Ear (AC average = 52 dB)**<br><br>1. According to WHO: 50–64 dB average = **Moderately severe hearing loss**.<br><br>**Determination of the Nature of Hearing Loss**<br>We compare the AC and BC thresholds in each ear:<br><br>If **BC** is also elevated (**>10 dB**), it indicates a sensorineural component.<br><br>If the **air-bone gap** (AC – BC) is **≥10 dB**, there is an additional conductive component.

	**Right Ear**<br><br>1. AC average = 65 dB, BC average = 54 dB → air-bone gap ≈ 11 dB.<br>2. BC > 10 dB (i.e., bone conduction is also elevated), and the gap ≥10 dB.<br>3. This pattern is consistent with **mixed deafness** (both a sensorineural and conductive component).<br><br>**Left Ear**<br><br>1. AC average = 52 dB, BC average = 40 dB → air-bone gap ≈ 12 dB.<br>2. BC > 10 dB, and the gap ≥10 dB again indicates a **mixed** component.<br><br>Based on our thorough analysis, we can conclude that **the degree of hearing in the patient's right ear is (E) severe hearing loss and the nature of the hearing loss in the right ear is (D) mixed deafness; and the degree of hearing in the patient's left ear is (D) moderately severe hearing loss and the nature of the hearing loss in the left ear is (D) mixed deafness.**
Report Interpretation	Below is a clear, step-by-step explanation of your hearing test results. I will use simple language to help you understand what each part means and how it may relate to your overall hearing health.<br><br>---<br><br>**1. Extract and Understand Key Information in the Report**<br><br>**Right Ear and Left Ear Hearing Thresholds**<br><br>1. Each ear has certain levels (measured in decibels, dB) showing how loudly sounds must be played for you to hear them.<br>2. In general, higher dB values mean you need louder sounds to hear, indicating more hearing loss.<br><br>**Air Conduction vs. Bone Conduction**<br><br>1. **Air conduction (AC)** measures how sound travels through the entire ear (outer, middle, and inner ear).<br>2. **Bone conduction (BC)** measures how sound travels directly to the inner ear, bypassing the outer and middle ear.

Degree of Hearing Loss

1. The report gives average hearing threshold numbers for your right ear (around 65 dB via air conduction) and left ear (around 52 dB via air conduction).
2. The bone conduction thresholds for the right ear (about 54 dB) and left ear (about 40 dB) tell us how well the inner ear alone is functioning.

---

2. Analyze the Hearing Loss in Different Frequency Bands

Low-Frequency Range (250–500 Hz)

- **What it usually means**: Low-frequency sounds include deep voices or humming noises.
- **Your results**: The chart suggests that even at lower pitches, you require louder levels than normal. This could be due to factors like fluid in the middle ear or age-related changes, although it varies by individual.

Mid-Frequency Range (1000–2000 Hz)

- **What it usually means**: These frequencies are very important for understanding speech clearly (consonants, vowels).
- **Your results**: There is a noticeable dip in your hearing at these mid frequencies, which may explain some trouble following conversations, especially in noisy environments.

High-Frequency Range (4000–8000 Hz)

- **What it usually means**: High pitches include birds chirping, certain speech sounds (like "s" or "th"), and higher musical notes.
- **Your results**: You also show a significant loss in the higher pitches, which is often linked to noise exposure (e.g., loud work environments) or natural aging processes.
- **Is it noise-induced?** If you have had a history of loud noise exposure (power tools, loud music, etc.), this could contribute to the greater drop in high frequencies.

Overall Trend

- Both low and high frequencies are affected, but the mid to high frequencies seem most impacted. That could mean you might find it especially difficult

to understand speech or pick up soft, high-pitched sounds.

---

3. Analyze the Auditory Conduction Pathways (Air vs. Bone)

Air Conduction (AC) Loss

- Your air conduction thresholds (the numbers in dB) are higher than normal, indicating that it takes louder sounds for you to hear.

Bone Conduction (BC) Loss

- Your bone conduction thresholds are also elevated, but they are somewhat better than your air conduction thresholds (although still not within normal range).

Possible Explanation

- Because **both** air and bone conduction thresholds are elevated, there is a **sensorineural component** (inner ear or nerve issue).
- Since **air conduction is worse than bone conduction** by a noticeable margin, there may also be a **conductive component** (outer or middle ear problem).
- When there is a difference of around 10 dB or more between air and bone conduction, we often call it a **mixed hearing loss** (a combination of both sensorineural and conductive factors).

---

4. Possible Causes of Hearing Loss

Noise Exposure

- Loud noise at work or during hobbies can damage the inner ear, especially at high frequencies.

Genetic Factors

- Some families have a pattern of hearing problems passed down through generations.

Age Factors

- As we age, high frequencies often drop first, known as

presbycusis.

Health Issues

- Middle ear infections, fluid buildup, or conditions like otosclerosis can cause conductive hearing loss (affecting how sound moves through the middle ear).

Acute Injuries

- Sudden loud blasts or trauma to the head/ear can cause immediate hearing damage.

---

5. Assess the Severity of the Hearing Loss

- **Right Ear**: The average threshold is around 65 dB, which suggests a **severe** level of hearing difficulty. You may struggle to follow normal conversation without hearing aids or extra volume.
- **Left Ear**: The average threshold is around 52 dB, indicating a **moderate to moderately severe** hearing loss. You might still hear some speech sounds, but it likely requires you to concentrate more, particularly in noisy settings.

Daily Impact

- In a quiet environment, you might catch speech if it is loud and clear.
- In a noisy environment, background noise can overwhelm softer speech, making conversations quite challenging.
- Using assistive devices (like hearing aids) and practicing good communication strategies (e.g., face-to-face talking, reducing background noise) can help.

---

6. Further Medical Evaluation or Treatment Recommendations

- Although your hearing test points to a **mixed hearing loss** (both sensorineural and conductive components), only a thorough exam by an ENT doctor can confirm the exact reasons.
- If there is any sign of a middle ear infection or fluid, medication or minor surgery may help the conductive part.
- For the sensorineural aspect, a hearing aid or other hearing device can help make sounds clearer and louder.
- It is advisable to protect your ears from further noise damage with earplugs

or noise-cancelling headphones in loud places.

Final Thoughts and Recommendations

Schedule an ENT Visit

- A medical professional can look for treatable causes in the outer or middle ear (like fluid, infection, or blockages).

Consider Hearing Aids

- Proper amplification can improve your quality of life, especially in social situations and at work.

Protect Your Ears

- If you're around loud sounds often, use hearing protection to prevent further damage.

Stay Informed

- Regular hearing check-ups (once a year or as recommended) will help track changes and adjust treatment if needed.

I hope this helps you understand your hearing test in simple terms. Remember, we want to help you hear better and feel comfortable communicating. If you have any more questions, don't hesitate to ask your audiologist or doctor for more information.

Professional advice	Below is a clear, step-by-step explanation and recommendation based on the data from your pure tone hearing report. We will walk through each step to help you understand what further tests may be needed, which treatments might help, and which lifestyle measures to adopt.<br><br>**1. Further Medical Diagnosis**<br><br>**Reasoning**<br><br>**Mixed Hearing Loss Indications**<br>1. The report shows both elevated air-conduction thresholds (outer/middle/inner ear pathway) and elevated bone-conduction thresholds (inner ear/nerve pathway).<br>2. There is a noticeable gap (about 10 dB or more) between air conduction (AC) and bone conduction (BC), suggesting a **mixed** loss (both conductive and sensorineural components).<br><br>**Why Further Tests May Be Needed**<br>1. To confirm the exact causes of the **conductive component** (e.g., middle ear fluid, eardrum issues, ossicular problems) and the **sensorineural component** (inner ear or auditory nerve damage), additional diagnostic steps can help:<br>1. **Otoscopy** (looking into the ear canal and eardrum)<br>2. **Tympanometry** (checks middle ear pressure/function)<br>3. **Acoustic Reflex Testing** (evaluates middle ear and neural pathways)<br>4. **Speech Audiometry** (assesses speech understanding in quiet and/or noise)<br><br>**Recommendation**<br>1. **ENT Consultation**: A visit to an Ear, Nose, and Throat (ENT) doctor is advisable. They can perform a thorough examination and rule out medical conditions such as ear infection, fluid in the middle ear, otosclerosis, or other treatable issues.<br>2. **Monitoring**: Since the sensorineural component often progresses slowly, regular hearing evaluations can track any changes and guide treatment adjustments.

2. Recommendation of Treatment and Intervention Programs

Reasoning

Conductive Loss Treatments

1. If there is fluid in the middle ear or infection, medications or minor surgeries (like myringotomy or middle ear procedures) may help.
2. Obstructions (e.g., earwax buildup or ear canal blockage) can be removed.
3. Otosclerosis (fixation of the stapes bone) may require surgical intervention to improve hearing.

Sensorineural Loss Treatments

1. **Hearing Aids**: Beneficial for sensorineural hearing loss; they amplify sound to help you hear more clearly, especially in daily conversations.
2. **Cochlear Implants**: Considered in more profound cases where conventional hearing aids provide limited benefit.

Mixed Loss

1. Addressing the **conductive** part can improve overall hearing, making the sensorineural aspect more manageable.
2. In many cases, a combination of **medical treatment** (e.g., surgery/medication for middle ear problems) **plus hearing aids** (for the sensorineural portion) yields the best results.

Which Treatment for You?

1. Because your test suggests **mixed hearing loss** (both conductive and sensorineural), you may benefit from:

    1. **Further medical assessment** to see if surgery or medication can fix or reduce the conductive portion.
    2. **Hearing aids** once any medical issues are resolved or stabilized, to amplify sounds for the sensorineural portion.

---

3. Lifestyle and Preventive Measures Recommendations

Reasoning

Noise Exposure

1. If you are around loud environments (e.g., factories, construction sites, loud music venues), use **earplugs** or **earmuffs** to protect what hearing you still have.

Healthy Hearing Habits

1. **Limit Headphone Volume and Duration**: Keep music or streaming sound at a moderate level. Take breaks if you listen for extended periods.
2. **Avoid Prolonged Loud Noise**: Continuous loud noise can speed up hearing damage.

Regular Checkups

1. Hearing changes can happen gradually. Having a **yearly or biannual hearing test** can detect any shifts early, especially important if there is a genetic predisposition or age-related decline.

Medication Awareness

1. Some drugs (often called "ototoxic") can affect hearing. Always consult with a doctor or pharmacist if you take medications for a long period.

If Aging or Genetic Factors Are Involved

1. Hearing naturally declines over time. Regular evaluations help track changes and ensure hearing aids or other treatments are updated as needed.

Professional Hearing Advice in Summary

Further Diagnosis

1. Consult an ENT to investigate the conductive portion of your hearing loss.
2. Conduct additional tests like tympanometry, speech audiometry, and an otological exam.

	**Treatment Strategy**<br><br>1. **Address Medical Causes** of conductive loss first (infection, fluid, or structural issues).<br>2. **Consider Hearing Aids** for the sensorineural portion and overall improvement once medical issues are managed.<br>3. If hearing aids provide limited benefit and the hearing loss is severe, ask your audiologist about cochlear implant evaluations.<br><br>**Lifestyle & Prevention**<br><br>1. Protect your ears from loud noises with earplugs or earmuffs.<br>2. Keep headphone volumes moderate and take regular breaks.<br>3. Get periodic hearing checkups to monitor any changes and adjust your treatment plan.<br><br>Following these steps will help clarify your hearing status, address any treatable causes, and ensure you receive the most effective long-term care for your hearing. If you have any questions or concerns, your audiologist and ENT specialist can provide more personalized guidance.

Supplementary Note 1

Data Collection Process and Inclusion/Exclusion Criteria (Based on Pure-Tone Audiometry Reports, Non-Human Subjects)

1.Sample Sources

Institutions: Second Affiliated Hospital of Zhejiang University School of Medicine (SAHZU) and Huiting (International) Hearing and Balance Center.

Location: Hangzhou, Zhejiang Province, China.

2.Data Collection Process

①Retrieval: Two trained graduate students (TTZ, QCF) retrieved pure-tone audiometry reports within the HIS (Hospital Information System) of two institutions by time period.

②De-identification: Hospital data anonymization scripts removed identifiable information such as names, ID numbers, phone numbers, and outpatient IDs, retaining only audiometric results and clinical diagnoses.

③Initial Screening: Reports were checked for inclusion of air-conduction and bone-conduction thresholds across all frequencies (125–8000 Hz); any missing frequency resulted in exclusion.

④Verification: One graduate student in informatics (MYX) cross-checked threshold completeness and de-identification effectiveness.

⑤Sample Lockdown: Ultimately obtained 140 fully de-identified reports with complete thresholds, spanning October 1, 2024, to December 31, 2024.

3.Inclusion Criteria

①Report generation date falls between October 1, 2024, and December 31, 2024.

②Includes air-conduction and bone-conduction thresholds for both ears at 125, 250, 500, 1000, 2000, 4000, and 8000 Hz.

③Accompanied by a clinical diagnosis issued by an otolaryngologist.

4.Exclusion Criteria

① Any frequency threshold is missing or recorded as "not tested."

② The report contains illegible handwriting or scanning noise rendering thresholds unreadable.

③ Subject age <3 years (unable to cooperate with behavioral audiometry).

④ Duplicate reports (multiple tests on the same subject on the same day; only the first test is retained).

5.Data Collection Period

On-site data capture and anonymization: January 2, 2025 to January 10, 2025.

Review and finalization: January 11, 2025 to January 15, 2025.

6.Ethics and Sharing

This study was approved by the Ethics Committee of SAHZU Medical School (Approval No. 2025-0027).

Supplementary References

1. CHART Collaborative. Reporting guidelines for chatbot health advice studies: explanation and elaboration for the Chatbot Assessment Reporting Tool (CHART). *BMJ* **390**, e083305 (2025).
